# Braitenberg Vehicles as Computational Tools for Research in Neuroscience

**DOI:** 10.3389/fbioe.2020.565963

**Published:** 2020-09-16

**Authors:** Danish Shaikh, Ignacio Rañó

**Affiliations:** Embodied Artificial Intelligence and Neurorobotics Laboratory, University of Southern Denmark Biorobotics Research Unit, Maersk Mc-Kinney Moller Institute, University of Southern Denmark, Odense, Denmark

**Keywords:** behavioral neuroscience, computational neuroethology, tropotaxis, navigation, Braitenberg vehicles

## Abstract

Valentino Braitenberg reported his seminal thought experiment in 1984 using reactive automatons or vehicles with relatively simple sensorimotor connections as models for seemingly complex cognitive processes in biological brains. Braitenberg's work, meant as a metaphor for biological life encompassed a deep knowledge of and served as an analogy for the multitude of neural processes and pathways that underlie animal behavior, suggesting that seemingly complex behavior may arise from relatively simple designs. Braitenberg vehicles have been adopted in robotics and artificial life research for sensor-driven navigation behaviors in robots, such as localizing sound and chemical sources, orienting toward or away from current flow under water etc. The neuroscience community has benefitted from applying Braitenberg's bottom-up approach toward understanding analogous neural mechanisms underpinning his models of animal behavior. We present a summary of the latest studies of Braitenberg vehicles for bio-inspired navigation and relate the results to experimental findings on the neural basis of navigation behavior in animals. Based on these studies, we motivate the important role of Braitenberg vehicles as computational tools to inform research in behavioral neuroscience.

## 1. Introduction

Behavioral neuroscience is the study of the structure and function of neural substrates in biological organisms with the goal of understanding the biological basis of behavior. Experimental approaches toward investigating neural mechanisms measure neural activity via voltage calcium imaging, neuron spike recording via electrodes as well as temporarily or permanently alter neuronal functioning by lesions, electrical or chemical stimulation and optogenetics. The theoretical approach toward understanding of brain function, i.e., computational neuroscience, utilizes mathematical modeling and computer simulation of neural structures to validate experimental data. While there is strong overlap and close interaction between experimental and theoretical neuroscience, there is clear consensus in the scientific community that interaction with the environment through a physical body is critical in fully understanding the role of neural substrates and mechanisms in behavior. The definition of behavior adopted here is as formulated by Levitis et al. (2009)— “the internally coordinated responses (actions or inactions) of whole living organisms (individuals or groups) to internal and/or external stimuli.”

### 1.1. Computational Aspects in Behavioral Neuroscience

While *in vivo* experimentation with live animal subjects to investigate the role of specific neural substrates via neural manipulation is commonplace in neuroscience, experimenting with an artificial brain is highly beneficial for investigating the computational and information processing aspects of behavior. The design and analysis of artificial brains that are inspired by or, where possible, that mimic biological neural structures and mechanisms must be embodied in a body that is situated in a virtual or real environment so that the behavior can be investigated within the context of a relevant ecological niche. This allows for controlling well-defined computational parameters that correspond to biological parameters that are relevant for the hypothesis under investigation. This approach is termed as embodied artificial intelligence (Pfeifer and Bongard, 2006), which embraces the idea that by designing and building artificial brains that are embodied within a robotic body, biological brain function and intelligence in general can be better understood. Designing such artificial counterparts of biological neural systems requires the use of computational modeling techniques, such as mathematical modeling used in computational neuroscience and neural networks to model functioning of larger neuroanatomical structures. When investigating neural mechanisms underlying perception, the motor system is abstracted away by using a robot with wheels. Where biological motor systems and their neural control is under investigation, robots that mimic the functional morphology are used. Floreano et al. (2014) have reported a broad overview of several studies at the intersection of robotics and neuroscience of invertebrate, vertebrate and primate behavior.

The advantage of such a cross-disciplinary approach is that it allows for testing coarse, high-level hypotheses, for example, regarding the role individual components in the sensorimotor pathway play in behavior generation and execution. This can inform the formulation of more low-level and finer hypotheses regarding specific neural sub-circuits and mechanisms to investigate neural functions in greater detail. This top-down approach to hypothesis formulation and testing can be more effective as well as efficient in understanding the neural basis of behavior. Using virtual or real autonomous agents, such as robots, situated in task-specific environments, in the experiment affords complete control over the subject, since the agent's parameters can be tightly controlled.

Animal behavior, although often appearing choreographed, is generated via a multitude of complex, dynamic and coordinated neural processes. The parameters of these processes are constantly being updated based on new sensory information, allowing animals to adapt to relevant changes in their environment. Sensory changes occur not only with changes in the environment, but also when the animal moves in and/or affects changes in (via specific motor actions) the environment. These changes affect the animal's perception of its world, which then affect its decisions regarding future movements and/or actions to be performed, which further affects the animal's subsequent perception of its world. Therefore, the animal's brain, body morphology and the environment together form a tightly coupled dynamical system. Understanding this holistic view of adaptive behavior in animals requires modeling the entire sensorimotor pathway, from perception to motor command generation.

The earliest attempts at modeling complete downstream sensorimotor pathways can be attributed to the eminent cyberneticist Valentino Braitenberg. He linked seemingly complex behavior to relatively simple sensorimotor circuits embodied as relatively simple vehicles (Braitenberg, 1984) in his seminal book *Vehicles: Experiments in Synthetic Psychology*. The vehicles he conceptualized display autonomous and complex behaviors in response to relevant stimuli. His conceptualization is governed by the “law of uphill analysis and downhill invention” which proposes that attempting to create the internal mechanisms underlying a behavior makes it easier to understand the same, rather than simply observing the behavior externally.

## 2. Braitenberg Vehicles as Models of Animal Behavior

Braitenberg vehicles in their simplest form comprise one or more sensor(s) such as, such as light, sound, chemical, etc. while the locomotor system is abstracted by two independent motorized wheels. The sensorimotor couplings are direct connections between the sensors and the motors. The nature and mechanism of the sensorimotor couplings dictates the behavior a given vehicle will display. Couplings can be ipsilateral where sensors and motors on the same side of the vehicle are connected together, or contralateral where sensors on one side are connected to motors on the opposite side of the vehicle. Couplings can also be inhibitory or excitatory. Inhibitory couplings imply that the stronger the stimulus as perceived by the sensor the weaker the excitation of the connected motor, while excitatory couplings imply that the stronger the stimulus as perceived by the sensor the stronger the excitation of the connected motor.

### 2.1. Tropotaxis With Braitenberg Vehicles

**Figure 1:**
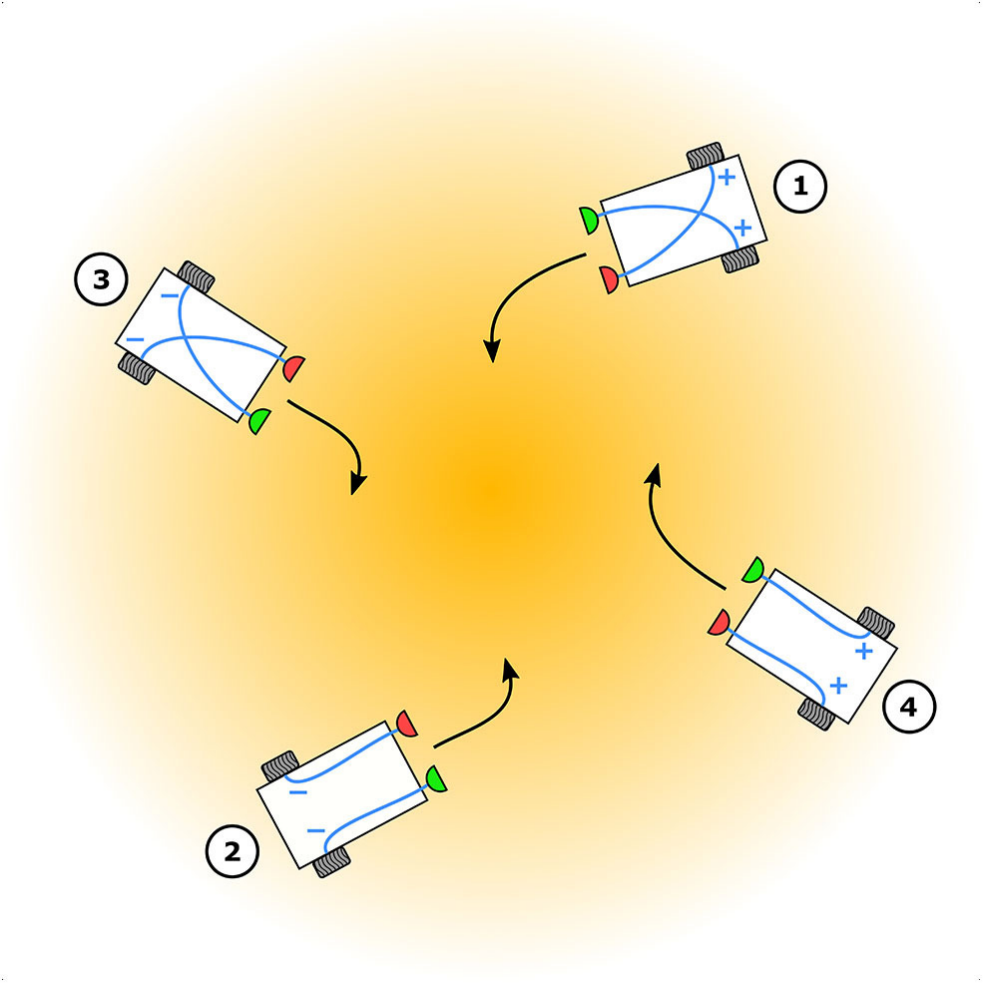
Elementary Braitenberg vehicle models for animal tropotaxis. Depending on the nature of the sensorimotor couplings (blue), “+” implying excitation and “−” implying inhibition, between the sensors (red and green) and the wheels (gray) vehicles 1 and 2 exhibit positive tropotaxis toward while vehicles 3 and 4 exhibit negative tropotaxis away from the stimulus source (orange) centered in the stimulus gradient.

Braitenberg vehicles serve well as models for animal tropotaxis (Figure 1), because they describe a holistic view of biological behavior by abstracting the underlying neural mechanisms via simple and direct sensorimotor couplings. This allows for relatively easy implementation in both simulation and as a real robot, and the behavior of the vehicle can be evaluated in real-world conditions. This makes them ideal as computational tools for testing relevant hypotheses in behavioral neuroscience. Although Braitenberg vehicles can model both positive tropotaxis (toward the stimulus source) as well as negative tropotaxis (away from the stimulus source), to the best of our knowledge, only the former has been investigated in the literature. This is likely because positive tropotaxis is the most interesting behavior as far as real-world robotic implementations are concerned. Robotic tropotaxis is a requirement in many practical and important applications of great utility to society, such as search-and-rescue robots responding to human voice, tracking gas plumes to localize potential gas leaks in buildings or tracking heat signatures to localize and map forest fires etc.

A vehicle with two sensors and two motors connected to wheels, and excitatory, contralateral couplings exhibits positive tropotaxis. Assuming that the stimulus source is to the left of the vehicle, the perceived stimulus at the left sensor is greater than that at the right sensor and thus the right motor runs faster than the left one. This makes the right wheel rotate faster than the left one and the vehicle thus turns left, toward the stimulus source. The closer this vehicle gets to the source, the more it accelerates because the perceived stimulus gets stronger for both sensors and both motors run faster, resulting in both wheels rotating faster. Ultimately, this vehicle will collide with the source. One can avoid this behavior by choosing inhibitory, ipsilateral couplings which also enable positive tropotaxis. Assuming again that the source is to the left, the perceived stimulus at the left sensor is greater than that at the right sensor and thus the left motor runs slower than the right one, making the left wheel rotate slower than the right wheel. The vehicle therefore turns left, toward the stimulus source. However, the closer this vehicle gets to the source the more it decelerates because the perceived stimulus gets stronger for both sensors and both motors thus experience weaker excitation, resulting in both wheels rotating slower. This vehicle eventually stops when it gets close enough to the source.

A vehicle with excitatory, ipsilateral couplings exhibits negative tropotaxis. Assuming a stimulus source to the left, the perceived stimulus at the left sensor is greater than that at the right sensor and thus the left wheel rotates faster than the right wheel. The vehicle therefore turns right, away from the stimulus source. Once the vehicle is facing away from the source, both the sensors perceive weaker stimulus levels and thus both wheels rotate slower. The vehicle thus decelerates as it moves further away from the source and eventually stops. If one chooses inhibitory, contralateral couplings the resulting vehicle also exhibits negative tropotaxis but in a different manner. Assuming a stimulus source to the left, the perceived stimulus at the left sensor is greater than that at the right sensor, making the right wheel rotate slower than the left one. The vehicle therefore turns right, away from the stimulus source. Once the vehicle is facing away from the source, both sensors perceive weaker stimulus levels and thus both wheels rotate faster. The vehicle thus accelerates and further it gets from the source, the faster it moves.

The following paragraphs present several case studies in the use of Braitenberg vehicles in neuroscientific hypothesis testing. The studies vary in the choice of biological organism under investigation as well as whether the hypothesis being tested is directly related to tropotaxis behaviors or to other related behaviors, such as obstacle avoidance.

### 2.2. Chemotaxis in Fruit Flies

Fruit flies (*Drosophilia melanogaster*) display stereotypical taxis behaviors when encountering relevant odors. Fruit fly larvae tend to localize and restrict themselves to the ripe parts of decaying fruits (Asahina et al., 2008), while adults localize food sources (Budick and Dickinson, 2006), and suitable sites for lying eggs (Joseph et al., 2009). Gomez-Marin et al. (2010) investigated chemotaxis behavior in larval and adult fruit flies using Braitenberg vehicles inspired by their sensory circuits and anatomical characteristics. One vehicle, modeling fruit fly larvae, had a laterally swinging head with two identical chemical sensors at its tip and inhibitory couplings to the motors on the ipsilateral sides. This vehicle turned toward the direction of orientation of the head, exhibiting positive tropotaxis. The inhibitory sensorimotor couplings caused the vehicle to move in the absence of odor stimulus similar to foraging behavior observed in fruit fly larvae. It also slowed down when nearing areas of high odor concentration and eventually stopped, similar to fruit fly larvae. Another vehicle, modeling fruit fly adults, had two separated but fixed chemical sensors with excitatory couplings to the motors on the contralateral sides. This vehicle steered toward areas of higher odor concentration. It accelerated past odor sources due to excitatory sensorimotor couplings and exhibited spiraling trajectories similar to those observed in flying flies encircling an odor source. Using these vehicles, the authors were able to test the hypothesis whether larvae and/or adults required bilateral comparisons of odor cues between the two chemical sensors to localize odor sources.

### 2.3. Chemotaxis in Cockroaches

The taxis behavior of the vehicles originally proposed by Braitenberg was driven solely by instantaneous values of the stimulus and did not account for its temporal dynamics. However, cockroaches localize food sources through smell via specialized olfactory receptor neurons termed as ON/OFF neurons that respond to temporal changes or gradients in perceived odor concentration (Hellwig and Tichy, 2016; Tichy et al., 2016). Freely moving cockroaches placed in a plume of relevant odor tend to follow a zigzag path toward the odor source, often deviating in and out of the plume (Willis and Avondet, 2005). This suggests that they may be sampling odor gradient information for steering. Zurro et al. (2019a,b) developed a Braitenberg vehicle model for biological chemotaxis inspired by the odor gradient processing by the ON/OFF neurons in the cockroach antennae. The vehicle implemented a linear weighted combination of instantaneous stimulus with temporal stimulus dynamics within the sensorimotor couplings. The vehicle had two identical, spatially segregated chemical sensors and ipsilateral, inhibitory sensorimotor couplings to enable positive taxis. The vehicle displayed zigzag-like trajectories when placed inside a simplified odor plume characterized by a Gaussian odor concentration distribution. Using this setup, the authors experimentally validated their hypothesis that linearly combining the instantaneous perceived stimulus and its temporal dynamics reduced oscillations in the vehicle's trajectories.

### 2.4. Phonotaxis in Lizards

Braitenberg vehicles have been applied extensively (Shaikh, 2012) to test hypotheses about the possible neural mechanisms underlying phonotaxis behavior in the Tokay gecko or *Gecko gekko*. The sensor model used here was a mathematical model of the peripheral auditory system of the animal, which is highly directional (Christensen-Dalsgaard and Manley, 2005) due to internal acoustical coupling between the animal's pinnae (Christensen-Dalsgaard and Manley, 2008). The authors developed a mobile robot which coupled binaural information from microphones about the spatial location of a sound source to motorized wheels via excitatory, contralateral connections. This sensorimotor coupling generated relatively successful phonotaxis behavior (Shaikh et al., 2009b). This confirmed their hypothesis that the traditional sense-plan-act paradigm, a deliberative approach to generating behaviors widely used in early research in mobile robot control, was not necessary for phonotaxis. The authors later incorporated decision models, whose outputs are time-dependent and delayed, into the sensorimotor couplings (Shaikh et al., 2009a). In biological terms, this corresponds to adding stimulus integration delays in the neural pathways from stimulus perception to motor signal generation. This made the phonotaxis behavior of the robot sensitive to the speed with which the sense-act loop was executed. Robot experiments confirmed their hypothesis that phonotatic performance with decision models that averaged over the stimulus samples should be relatively insensitive to the length of neural delay, while that with decision models that output the maximum of the stimulus samples should improve with decreased neural delay.

### 2.5. Phonotatic Tetrapod Locomotion in Salamanders

Salamanders move in an undulatory fashion characterized by lateral bending of the trunk (Roos, 1964; Daan and Belterman, 1968; Ritter, 1992), while maintaining the orientation of the head toward the direction of motion. Such head stabilization may be necessary to minimize oscillations in the head-centered reference frame for visual and auditory receptive fields. Without stabilization, self-generated head oscillations during phonotaxis would oscillate the auditory reference frame and generate oscillatory auditory cues regarding sound direction. This phenomenon was recreated by Shaikh et al. (2011) using the tetrapod robot *Salamandra robotica II* (Karakasiliotis and Ijspeert, 2009), with microphones located in its head, as a Braitenberg vehicle performing phonotaxis with oscillating auditory sensors. A central pattern generator model (Ijspeert et al., 2007) of the salamander motor system was used to control the gait of the robot. The lizard peripheral auditory system was used to generate auditory directional cues, which were coupled via excitatory contralateral connections to the central pattern generator model as drive signals. The authors used this robot to investigate whether stabilization of the head as observed in tetrapod locomotion in nature was necessary for successful taxis behavior (Shaikh, 2012). They were able experimentally demonstrate that successful phonotaxis was possible even with head oscillations.

### 2.6. Obstacle Avoidance in Bats

Echolocating bats must localize prey while simultaneously avoiding obstacles, such as trees. One such species, *Rhinolophus rouxii*, emits long, narrow-band ultrasound pulses during prey capture and listens for frequency and amplitude shifts in the echoes caused by moving prey (Schnitzler and Denzinger, 2011). However, echoes from stationary obstacles do not contain these shifts and this may help the animal detect and avoid obstacles (Lazure and Fenton, 2011). Vanderelst et al. (2015) developed a Braitenberg vehicle with two ultrasonic sensors that relied on such dynamics to perform obstacle avoidance behavior. They successfully tested the hypothesis that a bat could steer away from obstacles without requiring an internal three-dimensional model of the environment. Their Braitenberg controller steered a simulated bat left or right depending, respectively on whether the left or right ultrasonic sensor received the weakest echoes. It also steered the bat up or down depending, respectively on whether the sensor receiving the weakest echoes was currently pointing downwards or upwards. It used low-level cues, such as the interaural intensity difference and time delay of the first echo onset extracted from short echo pulses of 1 ms duration. This allowed the vehicle to react quickly when avoiding obstacles, similar to echolocating bats.

## 3. Discussion

The studies presented here cover two important sensing modalities for animal behavior—olfaction and audition. It is important to note that the robotics community has developed a number of analytical approaches for chemotaxis (see Chen and Huang, 2019 for a recent review) and phonotaxis (Huang et al., 1999; Bicho et al., 2000; Andersson et al., 2004; Zu et al., 2009; Zuojun et al., 2012; Hwang et al., 2014). Conventional robot chemotaxis approaches are either gradient-based (see Kowadlo and Russell, 2008; Ishida et al., 2012 for a review) or probabilistic and map-based, such as infotaxis (Vergassola et al., 2007), Bayesian inference (Wei Li et al., 2006), Kalman filtering (Blanco et al., 2013), particle filtering (Li et al., 2011), spatial memory-based behaviors (Grünbaum and Willis, 2015), Hidden Markov Models (Farrell et al., 2003), and kernel methods (Lilienthal et al., 2009; Reggente and Lilienthal, 2010). Gradient based approaches are computationally cheap and easy to implement but perform relatively poorly for realistic, intermittent odor plumes where computing gradients is challenging. Probabilistic and map-based approaches use successive odor sampling as well as wind velocity and direction information to update a plume distribution model which describes odor source location as a probability distribution. These approaches perform well when the environment and plume distribution model is accurate, which is non-trivial and cumbersome to develop. Conventional phonotaxis robots extract time-of-arrival-difference of sound at multiple microphones (typially 4–32) to compute sound direction. This requires precise, nanosecond-scale timing measurements which is non-trivial and computationally expensive.

There is no clear consensus on whether to use a biologically-inspired approach, such as Braitenberg vehicles or an analytical approach. One can argue that Braitenberg vehicles are relatively unoptimized approaches as compared to well-engineered analytical approaches, making an objective comparison of the two challenging, and thus one can expect the former to perform relatively worse than the latter. Hernandez Bennetts et al. (2012) argue that bio-inspiration for chemotaxis is of limited use since gas sensors and robot actuators have relatively slower responses than their biological counterparts. However, it is important to note that Braitenberg vehicles can serve as a model to test and refine both biological sensing and actuation models. Simply replicating animal movements without a deep understanding the underlying principles behind sensing and actuation in animals is a poor strategy which may worsen the performance of biologically-inspired approaches. We suggest that, generally speaking, analytical approaches are a better choice if the goal is simply to solve the problem of tropotaxis effectively while Braitenberg vehicles are an better choice if the goal is to understand underlying principles of biological tropotaxis. This does not imply that analytical approaches cannot serve to understand biological tropotaxis behavior. In fact, analytical approaches can reveal important details about the physics of sensing and actuation the animal must use to solve the tropotaxis problem.

## 4. Conclusions

Concepts such as synthetic psychology as envisioned by Valentino Braitenberg, cybernetics and embodied artificial intelligence have traditionally been disparate and have evolved separately into important research areas within themselves. These fields are highly relevant and complementary to behavioral neuroscience, and incorporating ideas and tools from these fields into behavioral neuroscience research can contribute significantly toward advancing our understanding of the neural basis of biological behavior.

We have motivated the use of Braitenberg vehicles as tools to test neuroethological hypotheses regarding animal behavior. We presented a brief review of several modeling studies which used Braitenberg vehicles to test hypothesis regarding a variety of behaviors in several different organisms. These studies indicate that Braitenberg vehicles can be useful tools in hypothesis testing in the investigation of perception systems, motor systems as well as sensorimotor coordination. By embodying a computational model of the neural structure of interest in a synthetic organism, such as a robot placed in a real and relevant environment, one can forgo efforts in modeling the environment or stimulus statistics, as these become available for free. Incorporating such embodied and situated computational models into the research methodology can inform hypothesis formulation and testing. This approach can guide behavioral neuroscience research toward novel avenues. However, one must be careful when formulating the hypothesis to be tested. Analysing the behavior of Braitenberg vehicles becomes correspondingly more cumbersome as the number of sensor modalities and the complexity of the structure of the sensorimotor couplings increase. This can make the formulation of a clear and testable hypothesis difficult, exacerbating the proper design of subsequent neuroscientific experimentation to validate the hypothesis in the biological organism.

### 4.1. Future Directions

Braitenberg did not originally consider plastic sensorimotor couplings, whose strength could be modified by rich sensorimotor experiences obtained via passive and active interaction between the vehicle and the environment. This opens new areas of investigation, such as exploring learning and memory in a closed-loop manner, in stark contrast to the open-loop approach taken in behavioral neuroscience experiments. Shaikh and Manoonpong (2019) have recently proposed a Braitenberg vehicle for phonotaxis with plastic sensorimotor couplings where the coupling strength is learned via the vehicle's interactions with obstacles through Hebbian learning. The study also revealed another important aspect of biological behavior briefly touched upon by Braitenberg in his book, which is its multisensory nature. Behaviors are often modulated by more than one sensory modality. For example, cockroaches navigating toward food sources using olfactory receptor neurons in their antennae often encounter walls along the way, which triggers a stereotypical wall-following behavior where tactile receptor neurons in the antennae help maintain a fixed distance from the wall. Braitenberg vehicles can help explore how can multiple different sensing modalities be coupled to motor systems to reproduce more complex behaviors.

## Author Contributions

All authors listed have made a substantial, direct and intellectual contribution to the work, and approved it for publication.

## Conflict of Interest

The authors declare that the research was conducted in the absence of any commercial or financial relationships that could be construed as a potential conflict of interest.
